# Association between Bell’s Palsy and Cardiometabolic Risks: An Age- and Sex-Matched Case–Control Study

**DOI:** 10.3390/jpm14020197

**Published:** 2024-02-10

**Authors:** Panitta Mueanchoo, Nualsakol Tepparak, Pensri Chongphattararot, Nannapat Pruphetkaew, Suwanna Setthawatcharawanich, Pat Korathanakhun, Thanyalak Amornpojnimman, Chutarat Sathirapanya, Pornchai Sathirapanya

**Affiliations:** 1 Department of Medicine, Faculty of Medicine, Prince of Songkla University, Hat Yai 90110, Songkhla, Thailand; butajung@hotmail.com (P.M.); cpensri@medicine.psu.ac.th (P.C.); ssuwanna@medicine.psu.ac.th (S.S.); kpat@medicine.psu.ac.th (P.K.); athanyal@medicine.psu.ac.th (T.A.); 2 Songkhla Rajanakarindra Psychiatric Hospital, Meung 90000, Songkhla, Thailand; nualsakol@hotmail.com; 3 Epidemiology Unit, Faculty of Medicine, Prince of Songkla University, Hat Yai 90110, Songkhla, Thailand; nannapat.pru@gmail.com; 4 Department of Family and Preventive Medicine, Faculty of Medicine, Prince of Songkla University, Hat Yai 90110, Songkhla, Thailand; schutara@medicine.psu.ac.th

**Keywords:** Bell’s palsy, diabetes, hypertension, lipid, metabolic syndrome

## Abstract

Background: Bell’s palsy is possibly an ischemic cranial neuropathy, although reactivation of herpes virus infection has been proposed. Methods: This was an age-and sex-matched and 1:2 case–control study enrolling Bell’s palsy patients during 2011–2021 in a university hospital to investigate the significant associations of cardiometabolic risks (CMRs) with Bell’s palsy. We analyzed the differences in waist circumference (WC), body mass index (BMI), systolic and diastolic blood pressures (SBP and DBP), fasting blood sugar (FBS), and lipid levels at 12 weeks post-Bell’s palsy with those of the controls by descriptive statistics (*p* < 0.05). The differences in means or medians of individual CMR values across the consecutive 10-year age intervals were analyzed by ANOVA *F*-tests and Kruskal–Wallis tests (*p* < 0.05). Results: A total of 140 cases and 280 controls were enrolled. Bell’s palsy patients had significantly higher WC, BMI, SBP, DBP, FBS, and triglyceride but lower high-density lipoprotein cholesterol (HDL-C) and low-density lipoprotein cholesterol (LDL-C). Moreover, high WC, elevated FBS (≥100 mg/dL), SBP (≥130 mmHg), and total cholesterol were significantly associated with Bell’s palsy cases by multivariable analysis. Only FBS in Bell’s palsy patients significantly elevated across consecutive 10-year age intervals. Conclusion: Screening and monitoring for CMRs, especially hyperglycemia, in every patient presenting with Bell’s palsy is essential despite initial normoglycemia, particularly in older-onset cases.

## 1. Introduction

Bell’s palsy is a common and considered benign cranial neuropathy. Reactivation of the latent herpes virus infection has been proposed as an etiopathogenesis. However, a higher prevalence of associated cardiometabolic risks (CMRs), such as hyperglycemia, hypertension, hyperlipidemia, metabolic syndrome (MS), etc., were reported in Bell’s palsy. The relation between these CMRs and Bell’s palsy has not been well understood [1]. Among the CMRs, hyperglycemia has been focused on by clinicians and researchers more than the others. Abnormal blood glucose homeostasis ranging from insulin resistance (IR), impaired oral glucose tolerance test (IGT), and impaired fasting blood sugar (IFBS) to overt diabetes mellitus (DM) was associated with Bell’s palsy with a wide range of reported frequencies. It was suggested that the patients presenting with Bell’s palsy might have an underlying abnormal glucose homeostasis (prediabetes state) or even unrecognized-DM-associated [2,3]. Hence, ischemic facial neuropathy was possibly an alternative etiopathogenesis of Bell’s palsy. In this regard, thrombosis of the vasa nervorum supplying the facial nerve in a long-standing abnormal glucose homeostasis state was attributed [4,5]. In the available studies, varying prevalence of IFBS, IGT, or overt DM reported in the patients presenting with Bell’s palsy depends on the different study settings, methods, study populations, and the length of follow-up time. A study by Yanagihara et al. reported DM in 7% of patients presenting with Bell’s palsy [6]. However, a prevalence as high as 31.5% was reported in a smaller sample size study (372 cases) [7]. Two retrospective hospital-based studies, including around one thousand cases per study, reported that 4.9% and 6% of Bell’s palsy patients had DM [8,9]. A study noted that while the overall prevalence of associated DM in Bell’s palsy patients was 11.4%, it increased to 16.8% in those aged ≥30 years [10]. This study suggested that patients with older-onset Bell’s palsy had a higher percentage of DM association. To now, there has been no natural cohort study to elucidate the actual timeline relation between the onset of Bell’s palsy and the development of DM. Whether Bell’s palsy is a preceding cranial neuropathy, a result of a hyperglycemic state, or both remains unrevealed. However, subclinical severe facial nerve degeneration, marked impairment of blink reflex, or concurrent symmetrical distal sensorimotor polyneuropathy detected by an electrophysiological study was more prevalent in patients presenting with Bell’s palsy associated with DM [11]. We conducted this cross-sectional, age- and sex-matched case–control study to assess the significant association between Bell’s palsy and CMRs such as IFBS, DM, hyperlipidemia, hypertension, and metabolic syndrome (MS) based on diagnostic criteria launched by the International Diabetes Foundation (IDF). The time point of 12 weeks after the onset of Bell’s palsy, when most of the cases recover favorably, was selected to assess the CMRs in this study to avoid the metabolic and biochemical interference from Bell’s palsy, its treatments, and its psychological impact. Based on the article by Eviston et al., the facial paralysis of Bell’s palsy reached its peak within 72 h after onset, and a total of 10 days for treatment with oral prednisolone was suggested. Thus, it can be presumed that the inflammation of the facial nerve lasts no longer than a few weeks [12].

## 2. Materials and Methods

### 2.1. Study Population, Design, Setting, and Sample Size

This was a 1:2, age- and sex-matched case–control study. We consecutively enrolled patients aged 15 years and up who were diagnosed with Bell’s palsy in Songklanagarind Hospital, the university hospital of Prince of Songkla University, from July 2011 to June 2021. Bell’s palsy was diagnosed by (a) an acute isolated unilateral peripheral facial paralysis without identifiable causes after the initial investigations including complete blood counts, random blood sugar, erythrocyte sedimentation rate, Venereal Disease Research Laboratory test (VDRL), anti-HIV, and chest radiography; and (b) the presenting facial weakness had a favorable outcome (House–Brackmann score, HBS ≤ 2) or complete recovery at 12 weeks after the onset. We enrolled the Bell’s palsy patients with varying degrees of severity of facial weakness at the presentation. One milligram (mg) per kilogram (kg) body weight of oral prednisolone for the initial 7 days, followed by a 10 mg/day taper-off regimen, was prescribed to the patients in this study. We did not start oral anti-herpes virus drugs concomitantly with the prescribed prednisolone. Eye shielding and application of eye lubricating gel on the affected eye to prevent exposure keratitis, self-massage, and facial muscle stretching to preserve the integrity of facial muscles were advised. No additional facial muscle rehabilitation procedures were applied at all. The controls were the yearly health checkup patients who had had no history of acquiring Bell’s palsy and visited the hospital during the same period as the study time. We randomly selected and matched the controls to cases by R program version 4.1.1 [13]. The number of study patients required for statistical significance calculated by the G*Power sample-size calculation website was 236 cases, of which 79 and 157 patients were cases and controls, respectively [14].

### 2.2. Terms and Definitions

The following terms and definitions were used in this study:

Impaired fasting blood sugar (IFBS) was a fasting blood sugar of 100–125 mg/dL.

Diabetes mellitus (DM) was a fasting blood sugar ≥ 126 mg/dL or being treated with glucose-lowering drug(s).

Hypertension (HT) was diagnosed when systolic blood pressure (SBP) ≥ 130 and/or diastolic blood pressure (DBP) ≥ 85 mmHg or being treated with blood pressure lowering drug(s).

Central obesity was defined by a waist circumference (WC) ≥ 90 cm in males or ≥80 cm in females according to the diagnostic criteria of metabolic syndrome (MS) for Asian populations endorsed by the IDF.

Metabolic syndrome (MS) was diagnosed based on the IDF-endorsed diagnostic criteria of MS [15,16]. The diagnosis required central obesity obligatorily, plus two or more of the following conditions: (a) TG ≥ 150 mg/dL; (b) HDL-C < 40 mg/dL (male) or <50 mg/dL (female); (c) SBP ≥ 130 mmHg or DBP ≥ 85 mmHg; and (d) FBS ≥ 100 mg/dL. Having been diagnosed with or receiving the treatments for one of the four conditions (a–d) above was considered to fit with the corresponding individual criteria.

### 2.3. Data Collection

We consecutively enrolled patients diagnosed with Bell’s palsy according to the definition mentioned. Baseline demographic data and previous CMRs, e.g., DM, HT, or hyperlipidemia (HLP), as well as the relevant treatment, were recorded. Those who had had any pre-existing diseases requiring regular treatment were advised to continue the treatment as usual during this study. We prescribed one course of oral prednisolone as described before and followed them at weeks 1, 3, 6, 9, and 12 when the facial paralysis of Bell’s palsy usually recovers favorably or completely. At week 12 of the follow-up, we recorded the body weight (BW), body mass index (BMI), waist circumference (WC), blood pressures, and results of blood tests related to the IDF’s diagnostic criteria of MS. The average of two blood pressure values, in which the first value was obtained after an initial 10 min rest and the second one was repeated after another 15 min rest, was recorded. We further added the blood tests for TC and LDL-C, which were not included in the IDF’s diagnostic criteria for MS. We further followed the patients until 24 weeks to ensure the diagnosis of Bell’s palsy. Since most of Bell’s palsy patients were reported to attain the most favorable or complete recovery at 24 weeks after the onset according to a literature review and the authors’ (P.S. and C.S.) previous study [7,17], if there was no favorable facial recovery appeared at 24 weeks, or the other differential diagnoses were suspected by repeated evaluations at any time point during the follow-ups, a brain imaging or other indicated tests would be performed to differentiate the causes of facial palsy. The cases in which the initial diagnoses of Bell’s palsy were finally revised were excluded from this study. The matched controls’ corresponding physical health parameters, blood pressures, and blood test results were collected for statistical analysis. They were advised to maintain their usual medications throughout this study.

### 2.4. Statistical Analyses

Descriptive statistics such as the Wilcoxon rank-sum test, *t*-test, or chi-squared test were used to analyze the significant difference in demographics, physical indices, and results of blood tests between cases and controls. Multivariable analysis was used to determine the significant association of the parameters indicated in the diagnostic criteria of MS endorsed by the IDF with Bell’s palsy, which was reported as an adjusted odds ratio (*p* < 0.05). We also analyzed the significant differences in the physical indices, blood pressures, and blood test results across the consecutive 10-year age intervals starting from <35, 35 to 44, etc., among the Bell’s palsy patients by chi-squared test, ANOVA *F*-test, and Kruskal–Wallis test (*p* < 0.05).

### 2.5. Ethical Considerations

The study protocol was reviewed and approved by the Ethic Committees of the Faculty of Medicine, Prince of Songkla University (EC No. 54-334-14-1-3). We strictly complied with the ethical regulations stated in the 1964 Declaration of Helsinki and its relevant guidelines to perform this research study. All the patients’ identifiable identities and personal information were completely anonymized. The data were analyzed aggregately to protect the study patients’ confidentiality. Signed consent to participate and publish this study results were obtained from all the study patients.

## 3. Results

From a total of 178 cases of peripheral facial palsy initially diagnosed as Bell’s palsy, 140 cases that met the study inclusion criteria were included for the statistical analyses. The excluded cases were 30 incomplete follow-ups, 5 revised diagnoses, and 3 cases with adverse effects from treatment. They were composed of 88 (62.9%) females. Their median (Q1, Q3) age was 52 (42, 62.2) years old. The onset of Bell’s palsy was prevalent during the rainy season in our area from June to November (63.7%). DM was a known pre-existing disease in 17 cases (12.1%), HT in 36 cases (25.7%), and HLP in 18 cases (12.9%). Other underlying diseases were two cases of hyperuricemia or gouty arthritis (1.4%), one case of coronary artery disease (0.7%), and two cases of thyroid disorders (1.4%). The current smokers and alcoholic drinkers were found in 17 (12.1%) and 5 (3.1%) cases, respectively. All the Bell’s palsy cases had a complete or favorable recovery (HBS ≤ 2) after the treatment with oral prednisolone at the 12-week follow-up.

We found significantly higher SBP, DBP, BW, BMI, WC, TG, and FBS levels but lower HDL-C and LDL-C levels among the Bell’s palsy cases (*p* < 0.05) by univariable analysis (Table 1). According to IDF’s diagnostic criteria of MS, we found that central obesity (WC ≥ 90 cm in males, or ≥80 cm in females), FBS ≥ 100 mg/dL, and SBP ≥ 130 mmHg were significantly associated with Bell’s palsy patients (*p* < 0.05). Additionally, multivariable analysis showed that TC, which was not included in the IDF’s proposed MS diagnostic criteria, was significantly higher in Bell’s palsy cases (Table 2).

By comparing the percentages of patients who had hyperglycemia, systolic or diastolic hypertension at the 12-week follow-up between Bell’s palsy cases and controls, the results showed that IFBS (100–125 mg/dL), DM (≥126 mg/dL), high SBP (≥130 mmHg), and both high SBP (≥130 mmHg) and DBP (≥85 mmHg) were significantly more prevalent in Bell’s palsy patients (Table 3). Moreover, the percentage of MS according to the IDF’s diagnostic criteria was found to be higher in Bell’s palsy cases (42.9%) than in controls (16.8%).

When we analyzed for the significantly elevated values of the cardiometabolic parameters among the Bell’s palsy patients across the 10-year age intervals, only FBS showed significant elevation across the age intervals (Table 4).

## 4. Discussion

This study found significantly higher BW, BMI, WC, SBP, and DBP in Bell’s palsy patients. Moreover, the blood tests showed significantly higher FBS and TG levels, whereas HDL-C and LDL-C were lower in Bell’s palsy cases (Table 1). These findings suggested the likelihood of the association of CMRs, as well as MS, with Bell’s palsy. Based on the current knowledge, central (or abdominal) obesity represented by high WC, which is the key feature required for the diagnosis of MS, is related to CMRs, e.g., prediabetes state (i.e., IGT and IFBS) or DM, HT, and HLP [18,19]. A population-based retrospective cohort study reported that general obesity (BMI ≥ 25 kg/m^2^) or central obesity (WC ≥ 90 cm in men or ≥ 85 in women) increases the risk of third, fourth, and sixth cranial palsies. Notably, the combination of general and abdominal obesity, rather than the individual type of obesity, further increased the risk of cranial nerve palsies [20]. Another retrospective cohort study also stressed that recovering from MS could reduce the risk of third cranial nerve palsy compared with chronic persistent MS [21]. The concurrent elevated gamma-glutamyl transferase, obesity, and MS were significantly associated with third cranial nerve palsy compared with the presence of only one or two conditions in a retrospective cohort study [22]. These findings signify the contribution of vascular pathogenesis of third and other oculomotor-control cranial nerve palsies. At present, there is no large cohort study evaluating the association between Bell’s palsy and MS or CMRs. According to our study, it is possible that patients presenting with Bell’s palsy have acquired MS or CMRs before the onset of facial paralysis. A population-based study supported our findings in that males, advanced age, residence in a location other than the capital and metropolitan cities, hypertension, and diabetes were significant risk factors for Bell’s palsy [23]. Therefore, careful screening and monitoring of CMRs is necessary for the prevention of future cardiovascular disorders. Although the reactivation of the latent herpes simplex virus (HSV) infection after primary infection of the virus was a commonly proposed pathogenesis of Bell’s palsy, several studies reported a higher prevalence of CMRs associated with Bell’s palsy patients, and therefore, ischemic facial neuropathy was a possible pathogenesis attributed [5,6,7,8,9,10,11,24,25,26,27]. Furthermore, a study reported that MS and CMRs overall retarded the recovery rate of facial weakness in Bell’s palsy as well [28]. Elevated glycosylated hemoglobin (HbA1c) levels >6.7% were reported to be significantly correlated with unsatisfactory recovery of Bell’s palsy in a study [5]. Nonetheless, another study argued that the severity of facial paralysis at presentation assessed by HBS, but not DM, affected the recovery of facial paralysis [29].

Among the CMRs, hyperglycemic state, either prediabetes or DM, has received more attention from the clinicians treating the patients presenting with Bell’s palsy. However, most of the currently available studies reporting the association between Bell’s palsy and the prediabetes state, DM, or MS were cross-sectional studies. [30,31,32,33,34]. With the heterogeneous study population, the settings, the designs, and the length of follow-ups, the reported prevalence of DM in Bell’s palsy patients varied from 2.5 to 11% [33,35,36]. Moreover, the definite timeline relation between the onset of hyperglycemia or other CMRs and that of Bell’s palsy has not been thoroughly studied. For this reason, it is uncertain how Bell’s palsy and these CMRs are related; either the detectable CMRs are causes or merely associated disorders of Bell’s palsy. However, it was suggested that the prediabetes state was possibly an associated disorder with Bell’s palsy, especially among those who have characteristics of MS or a personal history of diabetes risks [2,37,38]. Moreover, a study of the genetic association between HT and Bell’s palsy also reported that HT was a significant risk of Bell’s palsy at the genetic level, whereas DM and lipid disorders required further studies for confirmation [39]. Therefore, we suggest that the association of the prediabetes state or other CMRs with Bell’s palsy is possible and clinically crucial, requiring careful screening and close monitoring for the development of DM, other CMRs or cardiovascular diseases in the future.

In this study, we found a significantly higher percentage of IFBS (FBS 100–125 mg/dL), DM (FBS ≥ 126 mg/dL), isolated systolic HT (SBP ≥ 130 mmHg), and combined systolic and diastolic HT (SBP ≥ 130 mmHg and DBP ≥ 80 mmHg) in the patients presenting with Bell’s palsy (Table 3). Also, there were significant associations between Bell’s palsy and central obesity, FBS ≥ 100 mg/dL, SBP ≥ 130 mmHg, and hypercholesterolemia, as determined by the multivariable analysis (Table 2). The findings of central obesity, hyperglycemia, and systolic hypertension mentioned gave rise to the higher percentage of MS among the patients presenting with Bell’s palsy (42.9% vs. 16.8%) in this study. Our findings support the suggestion that the prediabetes state and MS should be considered in every patient presenting with Bell’s palsy. Furthermore, a study showed that an impaired 2 h oral glucose tolerance test (2 h OGTT) was significantly associated with Bell’s palsy patients who had normoglycemia at presentation [40]. This study emphasized the screening of the prediabetes state in all patients presenting with Bell’s palsy with 2 h OGTT despite normal FBS at the presentation. As 2 h OGTT is not included in routine initial evaluation of Bell’s palsy patients, we have no available data for analysis of the association of 2 h OGTT with Bell’s palsy. Since 2 h OGTT was considered a useful test for screening the prediabetes state, we suggest that it should be an additional test in evaluating Bell’s palsy patients, especially those who have diabetes risk or clinical features of MS. Future studies reporting impaired 2 h OGTT in Bell’s palsy cases are required to confirm the association.

It is noteworthy that only FBS levels significantly elevated across the consecutive 10-year older ages among the Bell’s palsy patients (Table 4). This finding could imply that the older-onset Bell’s palsy patients had a higher possibility of acquiring associated prediabetes or DM. While young-onset Bell’s palsy is possibly related to reactivation of the latent herpes virus infection which is a commonly proposed pathogenesis, facial nerve ischemia associated with prediabetes or eventual DM is more possible in older-onset cases.

In this study, we planned to evaluate the physical indices, the blood sugar, and other blood tests at 12 weeks after the onset of Bell’s palsy when most of the Bell’s palsy cases attained favorable or full recovery, and the prednisolone treatment had been completely terminated. At this time point, it could be presumed that the inflammation of the facial nerve completely resolved, and the effect of corticosteroid treatment on the blood test results, especially blood sugar, had been washed out too. We considered that the results of blood tests and blood pressure measurements obtained immediately at the presentation of Bell’s palsy may be interfered by the physical or psychological stress resulting from the concerns about the disfiguring facial appearance or progression of facial paralysis to hemiparesis as in a case of stroke. To now, there has been no evidence-based recommendation of the time point for performing the CMRs screening in patients presenting with Bell’s palsy. We strongly suggest longitudinal monitoring of CMRs in every patient acquiring Bell’s palsy, especially those who are at risk of cardiometabolic disorders.

### Strengths and Limitations

We considered that our study design of a 1:2 age- and sex-matched case–control study to evaluate the association of hyperglycemia, other CMRs, or MS with Bell’s palsy conducted at 12 weeks after the onset of Bell’s palsy would be more informative. However, the recommended time point for evaluation has not been suggested by clinical studies. Therefore, this may be a limitation of this study as the physical indices and/or blood test results may be interfered by dietary or physical activity factors experienced by the patients. Other limitations of this study were the small sample size and the retrospective design. We carefully excluded the cases that mimic Bell’s palsy at the presentations by careful history taking and neurological examination and longer follow-up time until 24 weeks after the onset of Bell’s palsy when most of Bell’s palsy patients recover completely to ensure the correct diagnosis, even though brain imaging was not performed in every case. Future prospective historical cohort studies are encouraged to elucidate the actual timeline relation between the development of every stage of hyperglycemia, HT, and HLP and the onset of Bell’s palsy.

## 5. Conclusions

Bell’s palsy possibly shares the pathogenesis of ischemic cranial neuropathy with other common diabetic cranial neuropathies. However, the actual timeline relation between various stages of hyperglycemia and the presentation of Bell’s palsy has not been specifically clarified. Based on the results of this study, the association between various CMRs and Bell’s palsy is likely, particularly hyperglycemia, among older-onset Bell’s palsy patients. Therefore, careful screening and close monitoring for the development of hyperglycemia among Bell’s palsy patients is crucial despite normoglycemia at the presentation.

## Figures and Tables

**Table 1:** Comparison of demographics, physical indices, and results of blood tests between Bell’s palsy cases and controls by univariable analysis.

	Case	Control	Total	Test Stat.	*p*-Value
Total	140	280	420		
Sex				Chisq. (1 df) = 0	1
Female	88 (62.9)	176 (62.9)	264 (62.9)		
Male	52 (37.1)	104 (37.1)	156 (37.1)		
Age (years) median (IQR)				Rank-sum test	0.929
	52 (42, 62.2)	53 (43, 61.2)	53 (42.8, 62)		
Age group (years)				Chisq. (11 df) = 0	1
(19,24)	3 (2.1)	6 (2.1)	9 (2.1)		
(24,29)	3 (2.1)	6 (2.1)	9 (2.1)		
(29,34)	7 (5)	14 (5)	21 (5)		
(34,39)	11 (7.9)	22 (7.9)	33 (7.9)		
(39,44)	18 (12.9)	36 (12.9)	54 (12.9)		
(44,49)	13 (9.3)	26 (9.3)	39 (9.3)		
(49,54)	23 (16.4)	46 (16.4)	69 (16.4)		
(54,59)	19 (13.6)	38 (13.6)	57 (13.6)		
(59,64)	18 (12.9)	36 (12.9)	54 (12.9)		
(64,69)	19 (13.6)	38 (13.6)	57 (13.6)		
(69,74)	5 (3.6)	10 (3.6)	15 (3.6)		
(74,79)	1 (0.7)	2 (0.7)	3 (0.7)		
SBP (mmHg)				Rank-sum test	<0.001 *
median (IQR)	136.5 (123, 150)	122 (111, 132)	126.5 (114, 140)		
DBP (mmHg)				Rank-sum test	<0.001 *
median (IQR)	80 (72, 85)	73 (65, 83)	76 (67.8, 83)		
Body weight (kg)				Rank-sum test	<0.001 *
median (IQR)	63 (55.1, 71)	57.7 (52.2, 66)	59 (53, 68)		
Height (m)				Rank-sum test	0.807
median (IQR)	1.6 (1.5, 1.6)	1.6 (1.5, 1.6)	1.6 (1.5, 1.6)		
BMI				Rank-sum test	<0.001 *
median (IQR)	25.2 (22.9, 27.7)	23.3 (21.5, 25.8)	23.9 (21.8, 26.3)		
WC (cm)				Rank-sum test	<0.001 *
median (IQR)	85 (79, 93.5)	80 (74, 86)	81.5 (76, 89)		
FBS (mg/dL) median (IQR)				Rank-sum test	<0.001 *
	99 (92, 111)	92 (87, 99)	94 (88, 102)		
TC (mg/dL)				*t*-test	0.051
mean (SD)	213.3 (43.5)	222.2 (43.3)	219.2 (43.5)	(404 df) = 1.96	
TG (mg/dL)					0.047 *
median (IQR)	116.5 (85, 167.2)	105 (73, 147)	109.5 (77, 151)	Rank-sum test	
HDL-C (mg/dL)				Rank-sum test	0.001 *
median (IQR)	50 (43.8,60)	55.2 (45.9,65.5)	53.3 (44.7,63.4)		
LDL-C (mg/dL)				*t*-test	<0.001 *
mean (SD)	133.8 (41.8)	151.9 (41.6)	145.8 (42.5)	(402 df) = 4.13	

* *p* < 0.05. Abbreviations: SBP, systolic blood pressure; DBP, diastolic blood pressure; BMI, body mass index; WC, waist circumference; FBS, fasting blood sugar; TC, total cholesterol; TG, triglyceride; HDL-C, high-density lipoprotein cholesterol; LDL-C, low-density lipoprotein cholesterol.

**Table 2:** The association of Bell’s palsy with the cut-off values of cardiometabolic parameters according to the diagnostic criteria of metabolic syndrome proposed by IDF by multivariable analysis.

Variables	OR (95% CI)	*p*-Value	Adj. OR (95% CI)	*p*-Value
BMI ≥ 23 vs. <23	2.31 (1.48, 3.59)	<0.001 *	1.41 (0.72, 2.75)	0.32
WC (cm) ^a^ Central obesity vs. normal	2.87 (1.86, 4.44)	<0.001 *	1.92 (1.02, 3.61)	0.04 *
SBP ≥ 130 vs. <130 mmHg ^a^	4.03 (2.53, 6.41)	<0.001 *	3.62 (2.04, 6.44)	<0.001 *
DBP ≥ 85 vs. <85 mmHg ^a^	1.32 (0.82, 2.11)	0.257		
FBS ≥ 100 vs. <100 mg/dL ^a^	4.72 (2.78, 8.01)	<0.001 *	4.68 (2.43, 8.99)	<0.001 *
TG ≥ 150 vs. <150 mg/dL ^a^	1.59 (0.98, 2.57),	0.058		
HDL-C, mg/dL ^a^ Low ^b^ vs. Normal	1.63 (1.02, 2.61)	0.041 *	1.25 (0.67, 2.32)	0.486
TC (cont. var.), mg/dL	0.9948 (0.9898, 0.9998)	0.039 *	0.9914 (0.9836, 0.9993)	0.03 *
LDL-C ≥ 100 vs. <100 mg/dL	0.47 (0.26, 0.86)	0.013 *	0.6 (0.23, 1.51)	0.273

^a^ Included in the diagnostic criteria of metabolic syndrome proposed by the IDF. ^b^ <40 mg/dL (male) or <50 mg/dL (female). * *p* < 0.05. Abbreviations: IDF, the International Diabetes Foundation; BMI, body mass index; WC, waist circumference; SBP, systolic blood pressure; DBP, diastolic blood pressure; FBS, fasting blood sugar; TG, triglyceride; HDL-C, high-density lipoprotein cholesterol; TC, total cholesterol; LDL-C, low-density lipoprotein cholesterol.

**Table 3:** Comparison of elevated FBS, SBP, DBP, and both SBP and DBP according to diagnostic criteria of metabolic syndrome proposed by IDF at 12 weeks following Bell’s palsy between cases and controls.

Variables	Case, *n* (%)	Control, *n* (%)	*p*-Value
FBS 100–125 mg/dL	44 (38.9)	48 (18.6)	<0.001 *
FBS < 100 mg/dL	69 (61.1)	210 (81.4)	
FBS ≥ 126 mg/dL	22 (16.3)	9 (3.4)	<0.001 *
FBS < 126 mg/dL	113 (83.7)	258 (96.6)	
SBP ≥ 130 mmHg	93 (66.4)	96 (34.3)	<0.001 *
SBP <130 mmHg	47 (33.6)	184 (65.7)	
DBP ≥ 85 mmHg	36 (25.7)	58 (20.7)	0.301
DBP < 85 mmHg	104 (74.3)	222 (79.3)	
SBP ≥ 130 and DBP ≥ 85 mmHg	34 (43.0)	47 (21.4)	<0.001 *
SBP < 130 and DBP < 85 mmHg	45 (57.0)	173 (78.6)	

* *p* < 0.05. Abbreviations: FBS, fasting blood sugar; SBP, systolic blood pressure; DBP, diastolic blood pressure; IDF, the International Diabetes Foundation.

**Table 4:** Analysis of the significant differences in SBP, DBP, BMI, WC, FBS, and blood lipid levels across the 10-year age intervals among the Bell’s palsy patients.

Age Intervals (Years)	<35	35–44	45–54	55–64	>=65	Total	Test Stat.	*p* Value
Total (n)	13	29	36	37	25	140		
Sex							Chisq. (4 df) = 5.17	0.27
female	9 (69.2)	20 (69)	25 (69.4)	23 (62.2)	11 (44)	88 (62.9)		
male	4 (30.8)	9 (31)	11 (30.6)	14 (37.8)	14 (56)	52 (37.1)		
SBP (mmHg)							ANOVA *F*-test (4, 135 df) = 2.07	0.089
mean (SD)	123.6 (18.6)	133.8 (14.4)	138.1 (17.5)	138.1 (20.1)	136.1 (13.2)	135.5 (17.3)		
DBP (mmHg)							ANOVA *F*-test (4, 135 df) = 2.21	0.071
mean (SD)	74.8 (10)	79 (10.2)	83.2 (11.3)	78.5 (9.8)	77.2 (10.1)	79.2 (10.5)		
Weight (kg)							Kruskal–Wallis test	0.275
median (IQR)	64.1 (54, 68)	64 (58, 73)	64 (56.1, 72.9)	63.1 (56.4, 69.7)	58 (54, 63)	63 (55.1, 71)		
Height (m)							ANOVA *F*-test (4, 133 df) = 1.33	0.264
mean (SD)	1.6 (0.1)	1.6 (0.1)	1.6 (0.1)	1.6 (0.1)	1.6 (0.1)	1.6 (0.1)		
BMI							Kruskal–Wallis test	0.435
median (IQR)	23.9 (21.8, 28.6)	25.2 (22.6, 27.7)	25.7 (24, 28.2)	25.4 (23, 27.7)	24.7 (21.3, 26)	25.2 (22.9, 27.7)		
WC (cm)							ANOVA *F*-test (4, 132 df) = 2.25	0.067
mean (SD)	79.7 (12.7)	83.6 (11.8)	87.7 (10.2)	89 (11.8)	87.2 (9.9)	86.4 (11.4)		
FBS (mg/dL)							Kruskal–Wallis test	<0.001 *
median (IQR)	88 (84, 92)	94 (88, 97.5)	102 (94, 115)	106 (98, 133)	106 (95, 113)	99 (92, 111)		
TC (mg/dL)							ANOVA *F*-test (4, 131 df) = 0.83	0.506
mean (SD)	209.2 (36.9)	217.6 (46.9)	218 (49.5)	215.9 (37.3)	199.2 (42.8)	213.3 (43.5)		
TG (mg/dL)							Kruskal–Wallis test	0.141
median (IQR)	81 (63, 120)	92.5 (81.8, 150)	132 (87, 187)	126 (102, 148)	117 (91, 169.5)	116.5 (85, 167.2)		
HDL-C (mg/dL)							Kruskal–Wallis test	0.707
median (IQR)	47 (42.8, 52)	50.3 (46.2, 56.1)	51.1 (46, 63.5)	50 (42.8, 60)	49 (42, 56.9)	50 (43.8, 60)		
LDL-C (mg/dL)							ANOVA *F*-test (4, 131 df) = 0.88	0.477
mean (SD)	137 (34.3)	139.6 (44.5)	130.1 (50.3)	139.4 (36.4)	121.6 (36)	133.8 (41.8)		

* *p* < 0.05. Abbreviations: SBP, systolic blood pressure; DBP, diastolic blood pressure; BMI, body mass index; WC, waist circumference; FBS, fasting blood sugar; TC, total cholesterol; TG, triglyceride; HDL-C, high-density lipoprotein cholesterol; LDL-C, low-density lipoprotein cholesterol,

## Data Availability

All the data and analysis methods are available from this published article.

## References

[1] Peitersen E. (2002). Bell’s palsy: The spontaneous course of 2,500 peripheral facial nerve palsies of different etiologies. Acta Otolaryngol. Suppl..

[2] Bosco D., Plastino M., Bosco F., Consoli A., Labate A., Pirritano D., Consoli D., Fava A. (2011). Bell’s palsy: A manifestation of prediabetes?. Acta Neurol. Scand..

[3] Johnson J.L., Duick D.S., Chui M.A., Aldasouqi S.A. (2010). Identifying prediabetes using fasting insulin levels. Endocr. Pract..

[4] Sazak Kundi F.C., Paksoy Z.B. (2023). Association of plasma atherogenic index with the severity and prognosis of Bell’s palsy. Acta Otolaryngol..

[5] Psillas G., Dimas G.G., Sarafidou A., Didangelos T., Perifanis V., Kaiafa G., Mirkopoulou D., Tegos T., Savopoulos C., Constantinidis J. (2021). Evaluation of Effects of Diabetes Mellitus, Hypercholesterolemia and Hypertension on Bell’s Palsy. J. Clin. Med..

[6] Yanagihara N., Hyodo M. (1988). Association of diabetes mellitus and hypertension with Bell’s palsy and Ramsay Hunt syndrome. Ann. Otol. Rhinol. Laryngol. Suppl..

[7] Zhao H., Zhang X., Tang Y.D., Zhu J., Wang X.H., Li S.T. (2017). Bell’s Palsy: Clinical Analysis of 372 Cases and Review of Related Literature. Eur. Neurol..

[8] Sathirapanya P. (1995). Bell’s palsy: A survey study of five-year hospital service. Chulalongkorn Med. J..

[9] Sathirapanya P., Limapichat K., Setthawatcharawanich S., Prabpal K., Sathirapanya C. (2003). Clinical characteristics of Bell’s palsy: A retrospective study of 978 cases in Songklanagarind Hospital. Songklanagarind Med. J..

[10] Kim M.H., Park S.Y. (2021). Population-based study and a scoping review for the epidemiology and seasonality in and effect of weather on Bell’s palsy. Sci. Rep..

[11] Stamatiou I., Papachristou S., Papanas N. (2023). Diabetes Mellitus and Bell’s Palsy. Curr. Diabetes Rev..

[12] Eviston T.J., Croxson G.R., Kennedy P.G., Hadlock T., Krishnan A.V. (2015). Bell’s palsy: Aetiology, clinical features and multidisciplinarycare. J. Neurol. Neurosurg. Psychiatry.

[13] R Foundation for Statistical Computing The R Project for Statistical Computing. R: A Language and Environment for Statistical Computing. https://cran.r-project.org/bin/windows/base/old/4.1.1/.

[14] Heinrich-Heine University Free Download G*Power. 2020, Version 3.1.9.7. https://g-power.apponic.com/download/.

[15] Zhu L., Spence C., Yang J.W., Ma G.X. (2020). The IDF Definition Is. Better. Suited for Screening Metabolic Syndrome and Estimating Risks of Diabetes in Asian American Adults: Evidence from NHANES 2011–2016. J. Clin. Med..

[16] Yousefzadeh G., Sayyadi A., Najafipour H., Sabaghnejad V., Pezeshki S. (2024). Comparing the association of two metabolic syndrome definitions, NCEP ATP III and IDF, with the risk of developing atherosclerotic cardiovascular disease: An analytical cross-sectional study. Endocrinol. Diabetes Metab..

[17] Sathirapanya P., Sathirapanya C. (2008). Clinical prognostic factors for treatment outcome in Bell’s palsy: A prospective study. J. Med. Assoc. Thai..

[18] Amouzegar A., Honarvar M., Masoumi S., Khalili D., Azizi F., Mehran L. (2023). Trajectory patterns of metabolic syndrome severity score and risk of type 2 diabetes. J. Transl. Med..

[19] Ishigaki Y., Hirase T., Pathadka S., Cai Z., Sato M., Takemura R., Ishida N. (2023). Prevalence of Metabolic Syndrome in Patients with Type 2 Diabetes in Japan: A Retrospective Cross-Sectional Study. Diabetes Ther..

[20] Choi D.D., Han K., Park K.A., Oh S.Y. (2022). Association of Obesity and Incidence of Third, Fourth, and Sixth Cranial Nerve Palsies. Am. J. Ophthalmol..

[21] Choi D.D., Park K.A., Han K., Oh S.Y. (2023). Dynamic Changes in Metabolic Status Are Associated with Risk of Ocular Motor Cranial Nerve Palsies. J. Neuroophthalmol..

[22] Kim J., Han K., Yoo J., Park K.A., Oh S.Y. (2022). Liver enzymes and risk of ocular motor cranial nerve palsy: A nationwide population based study. Neurol. Sci..

[23] Jeong J., Yoon S.R., Lim H., Oh J., Choi H.S. (2021). Risk factors for Bell’s palsy based on the Korean National Health Insurance Service National Sample Cohort data. Sci. Rep..

[24] Kim S.Y., Oh D.J., Park B., Choi H.G. (2020). Bell’s palsy and obesity, alcohol consumption and smoking: A nested case-control study using a national health screening cohort. Sci. Rep..

[25] Amalanathan S., Colbert K.R., Kumar C.S., Mathyalagen P. (2022). Clinical Prognostic Indicators in Predicting the Outcome in Patients with Bell’s Palsy: A Descriptive, longitudinal Study from Puducherry, South India. Indian. J. Otolaryngol. Head Neck Surg..

[26] Lee S.Y., Lim J.S., Oh D.J., Park B., Park I.S., Choi H.G. (2021). Increased risk of ischemic stroke in patients with Bell’s palsy: A longitudinal follow-up study using a national sample cohort. Auris Nasus Larynx.

[27] Kafle D.R., Thakur S.K. (2021). Evaluation of prognostic factors in patients with Bell’s palsy. Brain Behav..

[28] Jung S.Y., Jung J., Byun J.Y., Park M.S., Kim S.H., Yeo S.G. (2018). The effect of metabolic syndrome on Bell’s palsy recovery rate. Acta Otolaryngol..

[29] İnan S., Jafarov S. (2023). Prognostic role of neutrophil lymphocyte ratio and mean platelet volume in Bell’s palsy: Comparison of diabetic and non-diabetic patients. Braz. J. Otorhinolaryngol..

[30] Choi G.W., Yon D.K., Choi Y.S., Lee J., Park K.H., Lee Y.J., Park D.C., Kim S.H., Byun J.Y., Yeo S.G. (2023). Comparing the Clinical Manifestations of Bell’s Palsy between Pre-COVID-19 Pandemic and COVID-19 Pandemic Periods. J. Clin. Med..

[31] Ziv O., Hazout C., Goldberg N., Tavdi A., Zholkovsky A., Kordeluk S., El-Saied S., Dinur A.B., Ben-Zion J., Muhanna N. (2023). The Significance of Bell’s Palsy That Presents as Monocranial Versus Polycranial Neuropathy: A Case Series and Systematic Literature Review. Otol. Neurotol..

[32] Paolino E., Granieri E., Tola M.R., Panarelli M.A., Carreras M. (1985). Predisposing factors in Bell’s palsy: A case-control study. J. Neurol..

[33] Chen J., Yu Z., Zhou W., Cai H., Jin F., Hu J., Yu E., Xuan L. (2023). Effect of temperature and air pressure on the incidence of Bell’s palsy in Hangzhou: A distributed lag non-linear analysis. Sci. Rep..

[34] Zaccarelli-Marino M.A., Fonseca F.L.A., Gascón T.M., Filipini R. (2020). Profile of Overweight and Obesity in Children and Adolescents and Frequency of Type 2 Diabetes Mellitus and Glucose Intolerance: A Study in Public School in Brazil. Diabetes Metab. Syndr. Obes..

[35] Urban P.P., Forst T., Lenfers M., Koehler J., Connemann B.J., Beyer J. (1999). Incidence of subclinical trigeminal and facial nerve involvement in diabetes mellitus. Electromyogr. Clin. Neurophysiol..

[36] Stamboulis E., Vassilopoulos D., Kalfakis N. (2005). Symptomatic focal mononeuropathies in diabetic patients: Increased or not?. J. Neurol..

[37] Turgman J., Sarova-Pinhas I., Goldhammer J., Braham J. (1973). Prevalence of glucose intolerance in patients with Bell’s palsy. Isr. J. Med. Sci..

[38] Dillon B.R., Ang L., Pop-Busui R. (2024). Spectrum of Diabetic Neuropathy: New Insights in Diagnosis and Treatment. Annu. Rev. Med..

[39] Liu H., Sun Q., Bi W., Mu X., Li Y., Hu M. (2023). Genetic association of hypertension and several other metabolic disorders with Bell’s palsy. Front. Genet..

[40] Karagöz T., Bayir Ö., Çadalli Tatar E., Çakal E., Özdek A., Keseroğlu K., Şahin M., Korkmaz M.H. (2020). Prognostic role of homeostasis model assessment and oral glucose tolerance test in nondiabetic patients with Bell’s palsy. Turk. J. Med. Sci..

